# Trends in medication use by body mass index and age between 1988 and 2012 in the United States

**DOI:** 10.1371/journal.pone.0184089

**Published:** 2017-09-20

**Authors:** Arshdeep K. Randhawa, Jash S. Parikh, Jennifer L. Kuk

**Affiliations:** School of Kinesiology and Health Science at York University, Toronto, Canada; University College London, UNITED KINGDOM

## Abstract

**Objective:**

Whether the increase in prescription medication use over time differs by age and obesity status is unclear.

**Method:**

National Health and Nutrition Examination Survey (NHANES) between 1988 and 2012 was analyzed (n = 57,543).

**Findings:**

Increased medication use over time was seen in older individuals of all body mass index (BMI) classes, with the most prominent increase in those with obesity (p<0.001). For example, older men (≥65y) with obesity took 3.1 more medications between 1988 and 2012 versus 1.5 for normal weight older men. There were minimal differences in medication use over time in younger individuals. In men, the odds of taking antihypertensives, lipid-lowering medication, antidiabetics, and antidepressants increased with age, time and BMI wherein the association between age and medication use was magnified over time (age*time, p<0.05). In women, older women with overweight or obesity had a greater increase in the likelihood of antihypertensives and antidiabetics medication over time (BMI*time, p>0.05).

**Conclusion:**

Older individuals of all BMI classes may be driving the increase in medication use over time. However, the rise in the likelihood of taking cardiometabolic medications over time was generally not different between those with or without obesity in men with some increases seen in older women. Further research may be required to assess accessibility and barriers to medication use among certain demographics.

## Introduction

The use of prescription medications has increased over time in the United States [1,2]. This increase may reflect the development of new medications, the expansion of prescription drug coverage by insurance companies, and increased drug marketing by pharmaceutical companies. The greatest increase in medication use has been for obesity-related chronic conditions such as antihypertensives, antihyperlipidemics, antidiabetics and antidepressants [3–6]. In addition, there may be barriers to health care for individuals with obesity that may limit their access to medications. Indeed, the literature suggests that individuals with obesity face bias from health practitioners, have lower socioeconomic status and lack health insurance coverage [7,8]. Alternatively, the rise in medication use may be due to the increasing aging population who are also at elevated risk for these same chronic conditions [5,6,9]. Thus, it is unclear if the increase in medication use over time is due to the increasing prevalence of obesity, the aging population or whether there has been a systematic rise in medication use in these groups. Therefore, the objective of the present study is to examine the changes in the number and type of medication use by obesity and age between 1988 and 2012 in the United States.

## Methods

### NHANES

The National Health and Nutrition Examination Survey (NHANES) is a series of nationally representative cross-sectional surveys of civilians living in the United States. As a stratified, complex, multistage, probability-based survey, NHANES oversamples older adults, low-income individuals and certain racial/ethnic groups. The complete details of the study design and procedures are reported elsewhere [10]. Data for this study was obtained from the NHANES III (1988–1994, n = 33,994) and NHANES continuous surveys (1999–2000, n = 9,965; 2001–2002, n = 11,039; 2003–2004, n = 10,122; 2005–2006, n = 10,348; 2007–2008, n = 10,149; 2009–2010, n = 10,537; 2011–2012, n = 9,756).

Informed consent was obtained by all participants and ethics approval was obtained from the NHANES Institutional Review Board for NHANES III and the NCHS Research Ethics Review Board for the NHANES continuous surveys.

#### Sample size

Across all survey years, a total of 105,910 participants were interviewed. Analyses were based on the data collected from participants aged 18 years and older (n = 60,845). Participants were excluded additionally if data was missing on measured and self-reported body mass index (n = 3,201, education (n = 99) and prescription medication use (n = 100). The final sample size for complete case analysis was 57,543 persons.

#### Interview and examination measures

Questionnaires were used to assess age, sex, ethnicity (white or other), and education (≤ high school or > high school). Weight and height were measured by trained health technicians in a mobile examination center using standardized techniques and customized equipment. Body weight was measured on a digital weight scale (Mettler Toledo, Ohio, US). Standing height was measured in inches with a fixed stadiometer with a moveable headboard. Body mass index (BMI) was calculated as weight in kilograms divided by height in meters squared (kg/m^2^). Self-reported BMI was used for persons missing BMI measurement (NHANES III only, n = 1,696). Individuals were classified as underweight (BMI < 18.5 kg/m^2^), normal weight (BMI 18.5–24.9 kg/m^2^), overweight (25–29.9 kg/m^2^), and obese (BMI ≥ 30 kg/m^2^).

#### Prescription medication use

In all the NHANES surveys, information about prescription medication use was assessed during a household interview. Participants were asked if they had taken prescription medication over the past 30 days. Those who responded “yes” were asked to show the containers of the medication, and if unavailable, participants were asked to report the medication names. Medications were linked to a prescription medication database (Lexicon Plus) that includes all prescription medications classes. Medication classes for commonly used prescribed medications including antihypertensives, lipid-lowering medications, antidiabetics, antidepressants, analgesics, antibiotics, and sex hormones, were created using the prescription medication database. There were a maximum of 16 allowed medications reported in NHANES III and 20 in NHANES continuous surveys. For comparability, participants in the continuous surveys taking more than 16 prescription medications were recoded to 16 medications (in 15 men and 18 women) to be consistent with the maximum number of medications allowed in the NHANES III survey.

Antihypertensive drugs were coded to include agents for hypertensive emergencies, angiotensin converting enzyme, antiadrenergic agents, peripherally acting, centrally acting, beta-adrenergic blocking agents, calcium channel blocking agents, diuretics, peripheral vasodilators, antihypertensive combinations, angiotensin II inhibitors, vasopressin antagonists, aldosterone receptor antagonists, renin inhibitors, cholinergic agonists. Lipid-medication drugs were coded to include antihyperlipidemic agents, HMG-CoA reductase inhibitors, miscellaneous antihyperlipidemic agents, fibric acid derivatives, bile acid sequestrants, cholesterol absorption inhibitors, antihyperlipidemic combinations, miscellaneous metabolic agents. Antidiabetic drugs were coded to include antidiabetic agents, and miscellaneous metabolic agents. Antibiotic drugs were coded to include macrolide derivatives, miscellaneous antibiotics, penicillins, quinolones, sulfonamides, tetracyclines, aminoglycosides, lincomysin derivatives, glycyclines, antibiotics/antineoplastics. Sex hormone drugs were coded to include contraceptives, estrogens, gonadotropins, progestins, sex hormone combinations, miscellaneous sex hormones, gonadotropin releasing hormones.

### Statistical analysis

Descriptive statistics for the NHANES sample are shown as means ± SE and prevalence ± SE. All analyses were stratified for men and women separately. The differences between sample characteristics across survey years were assessed using one-way analysis of variance (ANOVA) with Tukey’s post hoc tests for continuous variables and chi-square tests for categorical variables. General linear modelling (GLM) was used to determine the association between BMI, age, and number of prescription medication taken over time. Multivariable logistic regression analysis was used to estimate the odds ratio (OR) of prevalent use of certain medication classes (i.e. lipid-lowering medications, antihypertensives, antidiabetics, antibiotics, analgesics, antidepressants and sex hormones). Main effects and interactions between BMI, age, and time on the number of prescription medication taken, or prevalent use of these medication classes were examined while adjusting for ethnicity and education. Normal weight was used as the reference category for BMI in both models.

Statistical hypotheses were tested using a two-sided α = 0.05 level. Analyses using NHANES III and NHANES continuous surveys separately were weighted to be nationally representative using SAS version 9.4 (SAS Institute, Cary, NC).

## Results

The participant characteristics for each survey year are shown in Table 1 for men and women separately. In general, the majority of the participants were white and highly educated. Across all survey years, 2.1±0.1% of the respondents were underweight, 33.2±0.4% were normal weight, 33.3±0.3% were overweight, and 31.4±0.4% had obesity. From 1988 to 2012, the proportion of individuals reporting the use of any prescription medication in the previous 30 days increased from 34.6±0.9% to 51.0±2.0% in men and 52.7±0.9% to 64.3±1.5% in women (Table 1).

**Table 1:** Baseline characteristics of US adults by NHANES surveys.

	NHANES III(1988–1994)	NHANES(1999–2000)	NHANES(2001–2002)	NHANES(2003–2004)	NHANES(2005–2006)	NHANES(2007–2008)	NHANES(2009–2010)	NHANES(2011–2012)
**Men**								
Sample size (n)	9106	2291	2505	2494	2507	2900	3043	2734
Age (years)	43.2 ± 0.4	42.9 ± 0.5	43.1 ± 0.5	44.2 ± 0.6	44.7 ± 0.9	44.7 ± 0.5	45.4 ± 0.5	45.4 ± 0.9
Ethnicity (% white)	76.5 ± 0.6	70.2 ± 1.1	72.3 ± 1.0	72.1 ± 0.6	72.5 ± 0.9	69.3 ± 0.9	67.9 ± 0.9	66.8 ± 1.0
Education (%, > HS)	42.3 ± 0.9	49.2 ± 1.4	54.0 ± 1.2	52.8 ± 1.3	55.1 ± 1.2	51.2 ± 1.2	56.5 ± 1.1	60.4 ± 1.3
BMI (kg/m^2^)	26.4 ± 0.1	27.6 ± 0.2*	27.8 ± 0.1*	28.0 ± 0.1*	28.5 ± 0.2*	28.3 ± 0.2*	28.6 ± 0.2*	28.4 ± 0.2*
Prescription (No.)	0.8 ± 0.02	1.0 ± 0.04*	1.2 ± 0.1*	1.5 ± 0.1*	1.5 ± 0.1*	1.6 ± 0.1*	1.7 ± 0.1*	1.6 ± 0.1*
Medication use (%)	34.6 ± 0.8	41.4 ± 1.5	43.3 ± 2.3	47.7 ± 1.7	46.0 ± 1.9	48.8 ± 1.7	49.6 ± 1.6*	51 ± 2.0*
*Antibiotics*	4.2 ± 0.3	4.0 ± 0.4	3.8 ± 0.4	3.3 ± 0.4	3.2 ± 0.5	3.2 ± 0.3	2.9 ± 0.4	2.7 ± 0.5
*Analgesics*	7.1 ± 0.4	9.1 ± 0.7	9.5 ± 0.7	13.2 ± 1.4	9.7 ± 1.0	10.5 ± 1.3	10.0 ± 0.8	9.3 ± 1.0
*Lipid medications*	2.1 ± 0.2	8.5 ± 0.6	10.4 ±1.1	13.2 ± 1.0	15.1 ± 1.0	16.9 ± 0.9	19.2 ± 1.2	18.1 ± 1.3
*Antihypertensives*	13.0 ± 0.7	17.8 ± 1.2	16.6± 1.1	22.2 ± 1.2	22.3 ± 1.4	24.0 ± 1.3	25.5 ± 1.4	25.4 ± 1.6
*Antidiabetics*	3.4 ± 0.3	4.4 ± 0.5	5.2 ± 0.4	5.7 ± 0.6	5.4 ± 0.6	7.4 ± 0.6	7.7 ± 0.7	8.5 ± 0.8
*Antidepressants*	2.0 ± 0.2	4.5 ± 0.7	6.6 ± 0.4	7.8 ± 0.6	6.9 ± 0.6	6.9 ± 0.7	7.6 ± 0.5	9.3 ± 0.9
**Women**								
Sample size (n)	10261	2593	2717	2687	2720	2976	3224	2785
Age (years)	45.0 ± 0.5	44.1 ± 0.5	43.8 ± 0.5	45.6 ± 0.6	45.9 ± 0.8	46.0 ± 0.6	46.3± 0.5	47.0 ± 0.9
Ethnicity (% white)	76.0 ± 1.2	68.2 ± 1.1	70.1 ± 1.0	71.7 ± 1.0	70.6 ± 1.0	69.5 ± 0.9	67.2 ± 0.9	65.3 ± 1.0
Education (%, > HS)	38.0 ± 1.2	49.0 ± 1.4	55.3 ± 1.2	54.9 ± 1.2	58.0 ± 1.2	55.3 ± 1.1	58.2 ± 1.0	65.2 ± 1.2
BMI (kg/m^2^)	26.2 ± 0.1	28.1 ± 0.3*	28.0 ± 0.2*	28.2 ± 0.2*	28.5 ± 0.3*	28.5 ± 0.2*	28.7 ± 0.1*	28.8 ± 0.2*
Prescription (No.)	1.2 ± 0.03	1.6 ± 0.1*	1.7 ± 0.1*	2.1 ± 0.1*	2.1 ± 0.1*	2.1 ± 0.1*	2.0 ± 0.1*	2.1 ± 0.1*
Medication use (%)	52.7 ± 0.9	58.7 ± 1.9	60.7 ± 1.8	62.9 ± 0.9	62.0 ± 1.3	63.5 ± 1.2	62.8 ± 1.9	64.3 ± 1.5
*Antibiotics*	6.5 ± 0.3	5.5 ± 0.7	4.8 ± 0.5	5.3 ± 0.4	5.6 ± 0.6	3.1 ± 0.3	3.3 ± 0.4	3.3 ± 0.4
*Analgesics*	11.5 ± 0.4	12.8 ± 0.8	15.6 ± 0.8	19.3 ± 1.3	12.6 ± 0.7	12.9 ± 1.1	13.2 ± 0.9	12.2 ± 1.3
*Lipid medications*	2.4 ± 0.2	6.3 ± 0.5	7.3 ± 0.6	10.6 ± 1.0	12.8 ± 1.3	15.0 ± 0.9	15.0 ± 0.7	17.5 ± 1.3
*Antihypertensives*	18.0 ± 0.5	19.9 ± 1.3	20.4 ± 1.4	24.6 ± 1.4	25.8 ± 1.4	26.5 ± 1.2	26.7 ± 1.6	27.5 ± 1.5
*Antidiabetics*	3.5 ± 0.3	4.2 ± 0.5	4.5 ± 0.5	6.3 ± 0.5	6.8 ± 0.7	7.3 ± 0.9	7.2 ± 0.4	7.4 ± 0.6
*Sex Hormones*	12.9 ± 0.6	19.2 ±1.2	21.5 ± 1.6	14.3 ± 0.8	12.9 ± 0.7	12.4 ± 1.2	10.3 ± 0.8	11.3 ± 1.1
*Antidepressants*	3.8 ± 0.2	10.1 ± 0.8	12.1 ± 0.9	14.4 ± 1.0	16.1 ± 0.7	17.2 ± 1.2	14.2 ± 1.1	16.9 ± 1.5

All the continuous values are presented as means ± SE and categorical values as prevalence (SE) %. HS = high school, BMI = body mass index. The values are weighted to be nationally representative.

* = significantly different from NHANES III (p<0.05).

### Association between BMI, and age with prescription medication use over time

**Fig 1:**
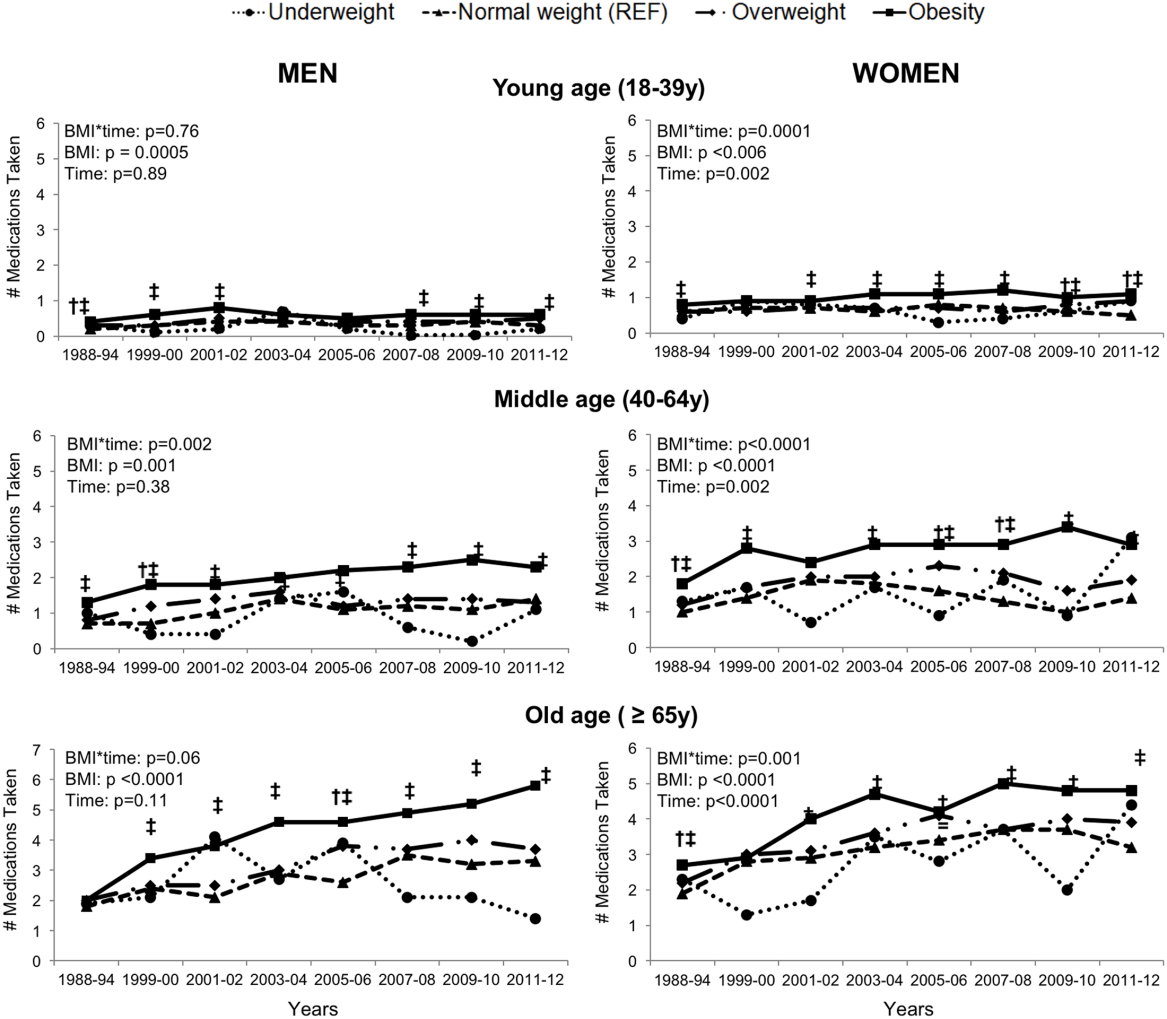
The number of prescription medications per BMI group over time in men and women by age category. BMI-related differences in prescription medication use were more apparent in older individuals and were magnified over time (age*BMI*time: P<0.001). BMI*time interaction is significant for each group (young, middle, old) in men and women (p<0.05). Significantly different from normal weight at that time point (* = underweight, † = overweight, ‡ = obesity; p<0.05). Mean values for the individual survey years were weighted and adjusted for ethnicity and education.

The relationship between age and BMI with the number of prescription medications taken over time is shown in Fig 1. In both men and women, there was a significant age*BMI*time interaction on number of prescription medications taken (p<0.001). Across all years, there were minimal differences in prescription medication use by BMI in young and middle aged individuals. Older individuals with obesity took more prescription medications than older individuals with normal weight in 1988, with the difference being magnified over time (p <0.001). Older men (≥65y) with obesity took 2.7 medications as compared to 1.8 medications for normal weight older men in 1988, and in 2012 took 5.8 and 3.3 medications, respectively (difference between obesity groups, 0.9 medications in 1988 versus 2.5 medications in 2012). Middle-aged men (40-64y) with obesity took 1.3 medications as compared to 0.7 medications for normal weight middle-aged men in 1988, and in 2012 took 2.3 and 1.4 medications, respectively (difference between obesity groups, middle aged men: 0.6 medications in 1988 versus 0.9 medications in 2012). Finally, younger men (18-39y) with obesity took 0.4 medications as compared to 0.2 medications for normal weight younger men in 1988, and in 2012 took 0.6 and 0.3 medications, respectively (difference between obesity groups, 0.2 medications in 1988 versus 0.3 medications in 2012).

### Odds of prevalent medication class use across time

**Fig 2:**
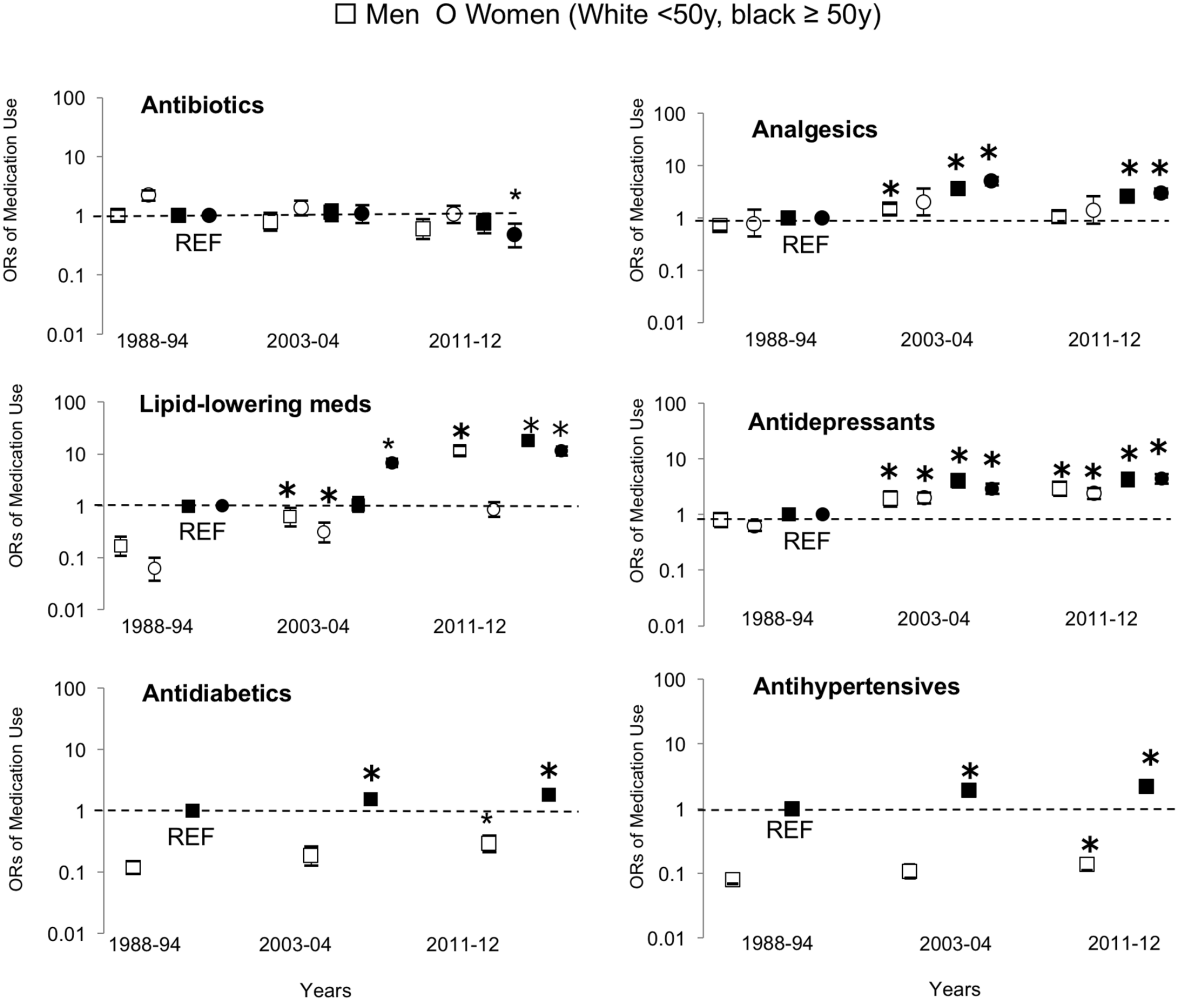
Odd ratios for prevalent medication class by age and time in men and women. The reference groups are older individuals (men and women separately) in the year 1988. For simplicity, only 3 of 7 years are shown. Models adjusted for ethnicity and education. *: Significantly different within age and sex group in 1988.

**Fig 3:**
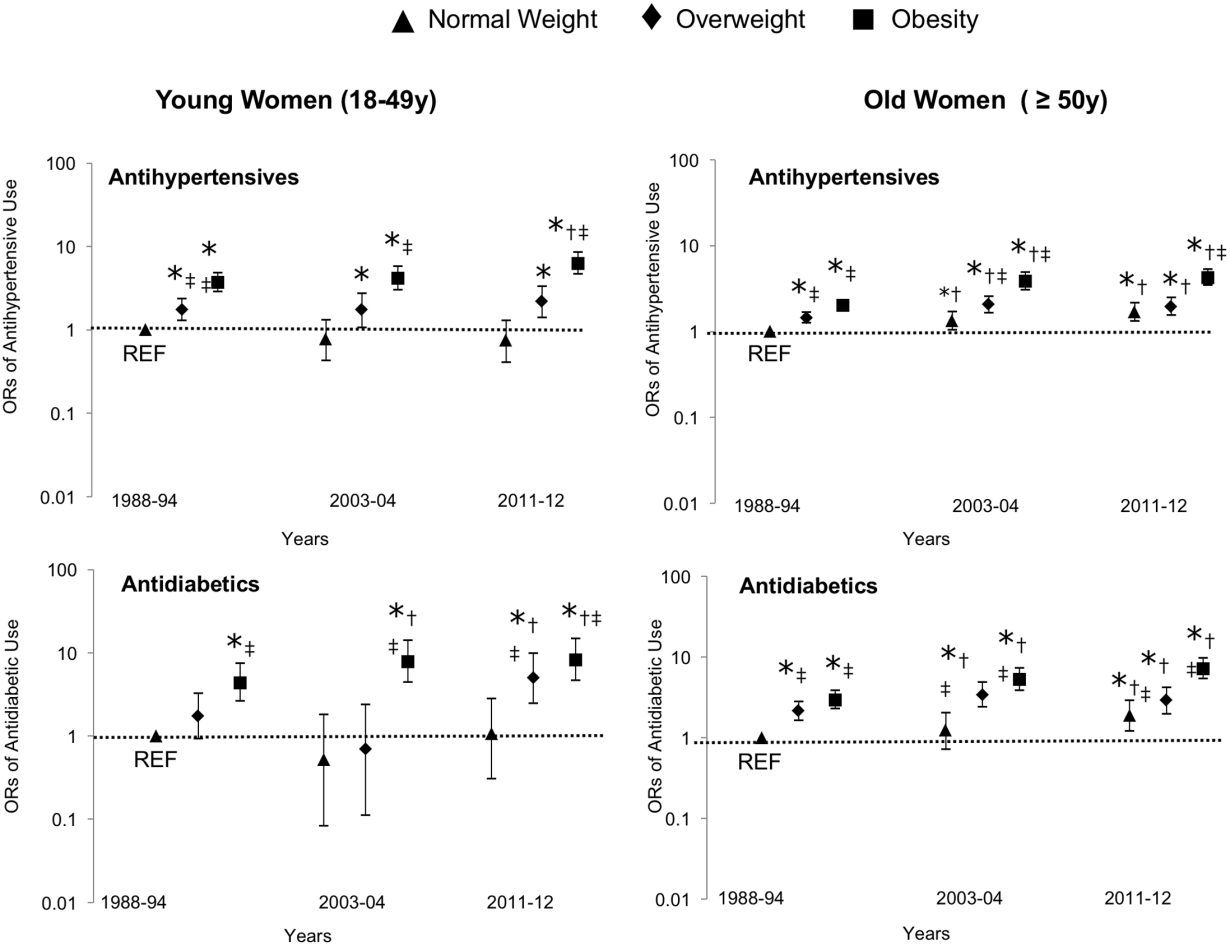
Odd ratios for prevalent antihypertensive and antidiabetics use in women by BMI, age and time. Both young and older women with obesity are more likely to be taking these medications over time, with the effect of time being significantly larger in older women. Models adjusted for ethnicity and education. Underweight sample was removed from the analyses. *: Significantly different from the reference group. †: Significantly different within the BMI category in 1988. ‡: Significantly different from the normal weight within the time period.

The OR for prevalent use of the most commonly prescribed medication classes is shown in Figs 2 and 3. For simplicity, only three survey cycles are shown to demonstrate trends over time (1988–1994, 2003–2004, and 2011–2012). There was no significant age*BMI*time interaction for prevalent medication class usage.

The likelihood of taking antidepressants (in both men and women), and lipid-lowering medications (in men) was higher with age and time (Fig 2, p<0.05) wherein there was a greater increase in the likelihood of taking these medications in older individuals over time than younger (age*time interaction; p<0.05). In younger women, generally there was no change in the use of lipid-lowering medications over time (time; p>0.05) however older women were more likely to take the medication over time (Fig 2, age*time interaction, p<0.05). There was no BMI*time interaction (p>0.05), but BMI was positively associated with the likelihood of taking antidepressants or lipid-lowering medications (p<0.05)

The likelihood of taking antibiotics was lower over time in both men and women (Fig 2, time; p<0.05). There was an age*time interaction in women (p<0.05) wherein the decreased in odds of antibiotic use was greater for older women over time (age; p<0.05). In men, there was no age*time interaction (age; p>0.05). No BMI differences were seen for the use of antibiotics for either men or women (p >0.05).

In men, there was a significant age*time interaction on the odds of analgesic use (Fig 2, p<0.05) The likelihood of analgesic use decreased post 2003–04 in both younger and older men wherein the decrease was greater in older men over time. In women, analgesic use remained consisted over time in younger and older women (p>0.05). BMI and age were both independently and positively associated with the odds of analgesics use in both sexes (p<0.05).

In men, the likelihood of taking antidiabetics and antihypertensives increased with age and time (p <0.05) wherein the increase was greater in older men over time (Fig 2, age*time, p<0.05). There were no BMI*time interaction in men for either medication (p>0.05). In women, age, BMI and time were independently and positively associated with the use antidiabetics, wherein women with obesity were more likely to take antidiabetics over time than normal weight women (Fig 3, BMI*time, p<0.05). For antihypertensives, there was also a BMI*time along with age*time interaction effect in women (Fig 3, p<0.05), wherein young and older women with obesity were more likely to be taking antihypertensives than normal weight women, with the rise in medication use over time being greater in older women and women with obesity.

The likelihood of taking sex hormones has increased over time (p<0.05) wherein the use of sex hormones decreased in older women over time (age*time interaction; p<0.05). For example, the odds ratios were 0.9 (0.8–1.1) for younger women (<50y) and 0.4 (0.3–0.5) for older women (≥50y) in 2012 compared to the reference group, older women in 1988. There was no change in the age with the use of sex hormones (p>0.05). In addition, BMI was negatively associated with sex hormone use (p<0.05) but there was no BMI*time interaction (p>0.05). Sex hormones use was not examined in men.

## Discussion

Our study extends previous research by demonstrating that older individuals of all BMI classes may have driven the increase in medication use over time. In particular, the increases in medication use over time were most prominent in older individuals with obesity, while there were minimal secular differences among younger individuals regardless of weight status.

We observe that older individuals were more likely to be taking antihypertensive, lipid-lowering and antidiabetic (men only) medications over time. This means that for a given BMI, an older individual was more likely to be taking a cardiometabolic medication in 2012 than 1988. The greater medication use may reflect increased medical insurance coverage, which may have made medications more affordable over time [2]. With improved access to health care and increased medication availability and effectiveness, there has also been an increase in life expectancy [11]. Thus, more individuals are surviving to older ages, and may further contribute to the rise in medication use. Though counter-intuitive, there is a negative association between obesity and mortality risk and improved survival from myocardial infarctions in older individuals with obesity, termed the “obesity paradox” [12,13]. In addition, to a greater likelihood of taking cardiometabolic medications, older individuals are taking a greater number of medications over time, particularly in those with obesity. This may reflect the many new pharmacological agents available for many of these chronic conditions [6]. For example, there are currently ten classes of medications available for type 2 diabetes compared to the lone pharmacological agent Metformin in the early 80s [14], contributing to the increased number of medications. Thus, the increase in medication use particularly in older individuals with obesity may reflect not only an increase in disease morbidity with aging but also increased lifespan over time.

Unlike age, obesity was not associated with preferentially greater increased odds of taking most classes of medication over time. Although obesity is associated with the increased likelihood of taking several types of medications, the general secular increases in medication use were similar in all BMI groups, with the exception being higher antihypertensive and antidiabetic medications use in women with obesity. This may reflect the greater number of physician visits reported by women about obesity in recent years [15]. In 2012, women were twice as likely to visit physicians’ office regarding obesity and associated risks than men [15]. Between 1988 and 2008, there was a substantial increase in hypertension awareness, treatment and control rates [16]. Similarly, there was a 20% increase in diabetes diagnoses in women between 1995 and 2012 [17]. Additionally, given the improvements in weight discrimination, there is a possibility that a higher proportion of women with obesity are seeking and receiving treatment for hypertension and diabetes over time.

For other medications, despite being at a higher risk for medical conditions, the preferential increase in medication use over time seen with older age is not apparent in those with obesity. This is surprising given that some research demonstrate that if treated, those with obesity are more consistent in using their prescribed medications than those with normal weight [4,18,19]. Thus, these results are more in line with the research suggesting that individuals with obesity face barriers to receiving health care, such as bias from health practitioners, low socioeconomic status and lack of health insurance coverage [7,8]. Alternatively, it is possible that the lack of secular rises in medication use may reflect the ‘healthier’ obesity phenotype over time [20]. For example, Gregg et al., [2005] demonstrated that the prevalence of high cholesterol and high blood pressure among those with obesity has decreased over time. Thus, the lack of secular rises in medication use in those with obesity may reflect a lower need or barriers to access.

The use of antidepressants has also increased over time and mainly among older individuals. Although, most cases of depression are diagnosed at a young age, the first episode can be experienced in older age which is referred to as late life depression [21]. Older individuals are more likely to develop depressive symptoms including functional & cognitive limitations, deficits in social support, loss of interest in living, solitude or hopelessness about the future. In addition, depression is associated with many medical conditions including cardiovascular diseases, Parkinson’s disease, and Alzheimer’s disease [22–24]. The increased use of antidepressants over time may reflect the changing attitudes toward seeking treatment or improved treatment for depression.

The secular trends we observed in the use of antibiotics, analgesics, and sex hormones may be in part due to the shift in policies regarding how certain medications are prescribed. For example, the use of antibiotics has decreased after the changes incorporated into Healthcare Effectiveness Data and Information Set (HEDIS) in 2006 which stated avoidance of antibiotic treatment in adults with acute bronchitis [25]. The stable use of analgesics over time may in part to individuals choosing to take over-the-counter (OTC) medications instead due to the increased awareness of the potential consequences such as overdose fatalities and risk for addiction [26,27]. Similarly, the decrease in sex hormones use over time in older women may reflect the increased awareness of the increased risk of coronary heart disease, breast cancer and stroke associated with sex hormone use from The Women’s Health Initiative Hormone Therapy Trial [28]. Therefore, despite an overall increased availability of medications, the use of certain medications have decreased due to policy changes over time in response to the increased awareness of adverse consequences associated with certain medication use.

Some strengths and limitations of the study are worth mentioning. One of the strengths of this study includes a large sample size of over 50,000 non-institutionalized individuals from the year 1988 to 2012 to document medication use within a nationally representative sample of the U.S. Further, the prescription medication data was obtained during an in-home interview by trained interviewers who reviewed a majority of the medication containers. This study is however, cross sectional, therefore causality cannot be implied.

In summary, our analysis demonstrates that increased medication use over time is mainly among older individuals of all BMI classes. Despite having a higher risk of developing chronic conditions in individuals with obesity, the rise in medication use over time was not different from normal weight. Our findings suggest that although there have been improvements in treating chronic conditions with medications, disparities by obesity status may continue to exist. Further investigation is needed to better understand the reasons for disparities in medication use.
